# Effects of single nucleotide polymorphism marker density on degree of genetic variance explained and genomic evaluation for carcass traits in Japanese Black beef cattle

**DOI:** 10.1186/1471-2156-15-15

**Published:** 2014-02-03

**Authors:** Shinichiro Ogawa, Hirokazu Matsuda, Yukio Taniguchi, Toshio Watanabe, Shota Nishimura, Yoshikazu Sugimoto, Hiroaki Iwaisaki

**Affiliations:** 1 Graduate School of Agriculture, Kyoto University, Sakyo-ku, Kyoto 606-8502, Japan; 2 National Livestock Breeding Center, Nishigo, Fukushima 961-8511, Japan; 3 Shirakawa Institute of Animal Genetics, Nishigo, Fukushima 961-8061, Japan

**Keywords:** Marbling score, Carcass weight, Japanese black cattle, SNP marker density, Linkage disequilibrium, Genomic evaluation, Genomic relationship matrix, Genetic variance estimation

## Abstract

**Background:**

Japanese Black cattle are a beef breed whose meat is well known to excel in meat quality, especially in marbling, and whose effective population size is relatively low in Japan. Unlike dairy cattle, the accuracy of genomic evaluation (GE) for carcass traits in beef cattle, including this breed, has been poorly studied. For carcass weight and marbling score in the breed, as well as the extent of whole genome linkage disequilibrium (LD), the effects of equally-spaced single nucleotide polymorphisms (SNPs) density on genomic relationship matrix (**G** matrix), genetic variance explained and GE were investigated using the genotype data of about 40,000 SNPs and two statistical models.

**Results:**

Using all pairs of two adjacent SNPs in the whole SNP set, the means of LD (*r*^*2*^) at ranges 0–0.1, 0.1–0.2, 0.2–0.5 and 0.5–1 Mb were 0.22, 0.13, 0.10 and 0.08, respectively, and 25.7, 13.9, 10.4 and 6.4% of the *r*^*2*^ values exceeded 0.3, respectively. While about 90% of the genetic variance for carcass weight estimated using all available SNPs was explained using 4,000–6,000 SNPs, the corresponding percentage for marbling score was consistently lower. With the conventional linear model incorporating the **G** matrix, correlation between the genomic estimated breeding values (GEBVs) obtained using 4,000 SNPs and all available SNPs was 0.99 for carcass weight and 0.98 for marbling score, with an underestimation of the former GEBVs, especially for marbling score.

**Conclusions:**

The Japanese Black is likely to be in a breed group with a relatively high extent of whole genome LD. The results indicated that the degree of marbling is controlled by only QTLs with relatively small effects, compared with carcass weight, and that using at least 4,000 equally-spaced SNPs, there is a possibility of ranking animals genetically for these carcass traits in this breed.

## Background

Most economically important traits in beef cattle, including carcass traits, are controlled by many quantitative trait loci (QTLs), which usually have relatively small individual effects. For such traits, genomic evaluation (GE) and selection (GS), as proposed by Meuwissen *et al.*[1], is expected to chase the QTLs simultaneously using single nucleotide polymorphism (SNP) markers, given that at least one SNP is in linkage disequilibrium (LD) with each QTL. In concept, successful GS is expected to accelerate genetic improvement by reducing the generation interval and increasing the accuracy of genetic evaluation [2].

The recent development of various SNP chips has enabled high-throughput genotyping and allowed animal breeders to study and conduct GE and GS. By simulating 50,000 genome-wide high-density biallelic markers like SNPs, VanRaden [3] showed better performance of best linear unbiased prediction (BLUP) using a genomic relationship matrix (**G** matrix) relative to that using an additive relationship matrix (**A** matrix), based on pedigree information [4]. In dairy cattle, GS has already been adopted in some countries and is an effective method for increasing the rate of genetic improvement. In beef cattle, on the other hand, its adoption has been slower, because the accuracy of the genomic estimated breeding value (GEBV) is much lower because of less availability of sires with highly accurate results in progeny tests.

Habier *et al.*[5] proposed the use of lower-density and equally-spaced SNP panels for effective GE, irrespective of trait. If such SNPs can explain substantial proportions of genetic variations in carcass traits and be almost as effective as higher-density panels in evaluating GEBVs, their lower cost would make them useful, especially in beef breeding females. Traits that are measured after slaughter, as well as those that are difficult or expensive to record, are also traits for which GS could substantially improve genetic gain. However, for carcass traits, including degree of marbling in beef cattle, the effects of differing densities of SNPs used to estimate genetic variance and GE have been poorly studied.

Japanese Black cattle are the primary breed of Wagyu, which are the modern native beef cattle in Japan, and are well known for meat qualities such as marbling. This breed has also been distributed for beef production in North and South America and Australia. In Japan, native Japanese cattle were crossed with British and Continental breeds during an approximately 10 year period in the early 1900s, and then, under a completely closed breeding system, the four breeds of Wagyu, including the Japanese Black, were fixed through strict selection over many years [6]. Moreover, since the relaxation of beef import restrictions in Japan in 1991, beef quality traits such as marbling have received more emphasis in the domestic production of the Japanese Black. In the same year, genetic evaluation of carcass traits using the mixed model methodology began [7]. These factors have led to intensive use of a low number of sires with high predicted breeding values for meat quality, and consequently a sharp decline in effective population size [8].

The accuracy of GE depends on the extent of LD between SNP markers and QTLs, the number of animals with phenotypes and genotypes in the reference population, the heritability, and the distribution of QTL effects for the trait [9]. The first of these factors is closely related to effective population size, and the density of SNP markers used that can be under the control of animal breeders. In this study, effects of density of equally spaced genome-wide SNPs on genetic variance explained and GE were investigated for carcass traits in beef cattle, using Japanese Black data and assuming two statistical models.

## Results and discussion

### Extent of linkage disequilibrium

**Figure 1:**
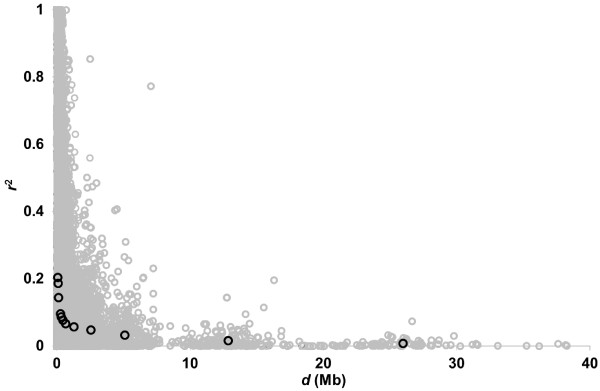
**Change in mean of *r***^***2***^**against mean of *d *** **(black circles), together with all values of *r***^***2***^**(gray circles).**

For the extent of LD, summary statistics of the squared correlation (*r*^*2*^*)* and the distance (*d)* for all pairs of two adjacent SNPs in each SNP set are presented in Table 1. Figure 1 depicts the changes in means of *r*^*2*^ and *d*, together with all values of *r*^*2*^. With all available SNPs, the means of *r*^*2*^ and *d* were 0.204 and 0.07 Mb, respectively. When the number of SNPs used was decreased to 20,000, 10,000, 8,000, 6,000 and 4,000, the average *r*^2^ values became 0.144, 0.096, 0.086, 0.077 and 0.066, respectively, and the corresponding means of *d* were 0.13, 0.26, 0.33, 0.44 and 0.65 Mb in turn. With all the SNPs, the means of *r*^*2*^ at ranges 0–0.1, 0.1–0.2, 0.2–0.5 and 0.5–1 Mb was 0.22, 0.13, 0.10 and 0.08, respectively, and 25.7, 13.9, 10.4 and 6.4% of the *r*^*2*^ values exceeded 0.3, respectively.

**Table 1:** Extent of linkage disequilibrium and distance between two adjacent SNPs and correlations for elements of G matrices

**No. of SNPs selected**	***r***^**2**^		***d *** **(Mb)**		Correlation*		
	**Mean**	**SD**	**Mean**	**SD**	***r***_***D***_	***r***_***N***_	***r***_***A***_
100	0.008	0.011	25.98	3.15	0.61	0.51	0.59
200	0.017	0.027	12.86	1.95	0.74	0.64	0.72
500	0.032	0.060	5.10	1.03	0.82	0.79	0.86
1,000	0.048	0.077	2.55	0.62	0.89	0.88	0.92
2,000	0.057	0.093	1.27	0.39	0.94	0.94	0.96
4,000	0.066	0.108	0.65	0.65	0.97	0.97	0.98
6,000	0.077	0.121	0.44	1.03	0.98	0.98	0.99
8,000	0.086	0.136	0.33	0.66	0.99	0.99	0.99
10,000	0.096	0.151	0.26	0.45	0.99	0.99	0.99
20,000	0.144	0.215	0.13	0.29	0.99	0.99	0.99
30,000	0.187	0.261	0.09	0.24	0.99	0.99	0.99
38,502	0.204	0.275	0.07	0.20	-	-	-

*Correlations between the diagonal (*r*_*D*_), upper triangular (*r*_*N*_) and all the elements (*r*_*A*_) of two **G** matrices constructed using a given SNP subset and all available SNPs.

Investigating the overall average of *r*^*2*^ using 2,670 SNPs for eight breeds, including Japanese Black cattle, McKay *et al.*[10] reported that the average for 65 Japanese Black cattle was approximately 0.58 for all SNP pairs ≤ 1 kb apart, and 0.07 for all SNP pairs ≤ 2 Mb apart. In the current analysis, the mean *r*^*2*^ for pairs of two adjacent SNPs, ≤ 1 kb–≤ 2 Mb apart, was 0.81–0.20, and most of the average *r*^*2*^ values obtained using all the SNPs in each given distance range were higher than those reported for the eight breeds (data not shown). Furthermore, in Dutch and Australian Holstein-Friesian, Australian Angus and New Zealand Friesian and Jersey cattle, using about 3,000–7,000 SNPs, the average *r*^*2*^ of 0.35 for inter-marker distances of 0–0.01 Mb declined to 0.22 for 0.02–0.04 Mb and 0.14 for 0.04–0.1 Mb [11]. As shown in Figure 1, a largely similar pattern of decreasing LD was observed with the current data for Japanese Black cattle. However, it should be noted that most samples used in these previous studies were from a subpopulation, especially in representative dairy and beef breeds, or many small-scale families of the breed, including sires in some cases, which would be a factor responsible for constructed haplotype blocks in the population. In contrast to this, the samples used in the current study were collected at two large-scale meat markets in Japan, to which fattened Japanese Black animals are sent from all over Japan. Therefore, samples used in this study are considered to reflect the effective size and LD extent of the national Japanese Black population.

Using the genotype data from 18,098 SNPs with minor allele frequencies (MAF) greater than 10% for 25 artificial insemination (AI) sires of Brazilian Gyr dairy cattle, Silva *et al.*[12] found that means of *r*^*2*^ and *d* for two adjacent SNPs ranged from 0.24–0.17 and from 0.12–0.18 Mb, respectively, at the autosome-wide level. In the current study of Japanese Black cattle, also with a relatively low effective population size, the mean *r*^*2*^ was nearly the same, but the mean *d* was about half that at the autosome-wide level (data not shown). Silva *et al.*[13] also observed that at ranges 0–0.1, 0–0.2, 0–0.5 and 0–1 Mb, mean *r*^*2*^ was 0.20, 0.18, 0.14 and 0.11, respectively, and that the proportion of SNP pairs exhibiting *r*^*2*^ higher than 0.3 was 22.9, 19.7, 14.1 and 9.5% for the same ranges, respectively. In this study, for the same ranges, mean *r*^*2*^ was 0.22, 0.21, 0.20 and 0.20, respectively, and the proportion of SNP pairs was 25.7, 24.1, 23.6 and 23.6%, respectively. From the current results, it is therefore likely that the extent of LD between more distant SNPs is relatively higher in Japanese Black cattle.

In addition, calculating the *r*^*2*^ of all possible SNP pairs by chromosome, from more than 30,000 SNPs distributed genome-wide, the extent of LD and the structure of haplotype blocks were examined for 19 breeds, including Indicus, African and the composite cattle, in addition to some dairy and beef breeds [13], and for Angus, Charolais and crossbred beef cattle [14]. Also, for Nellore cattle, whole genome LD was investigated using about 450,000 SNPs [15]. Yan *et al.*[16], using 632 maize lines genotyped for 1,229 SNP markers, demonstrated an increase in *r*^*2*^ values between the markers, especially between closer SNP pairs, with an increasing MAF threshold and an increase, particularly between more distant pairs, with decreasing sample size. The MAF threshold we used was smaller than those in previous studies [10-15], and sample size in the current study was larger than those for most of used in these studies. In this study, we only calculated the *r*^*2*^ of all pairs of two adjacent SNPs, avoiding a heavy computational burden. When our limited results were compared with the results of the previous studies, we found that, while the extent of whole genome LD in Zebu cattle, such as the Nellore, was relatively low, whole genome LD in the Japanese Black was likely to be higher than, or equal to, the whole genome LD in Angus, which was higher than in Charolais.

### Change in the genomic relationship matrix

**Figure 2:**
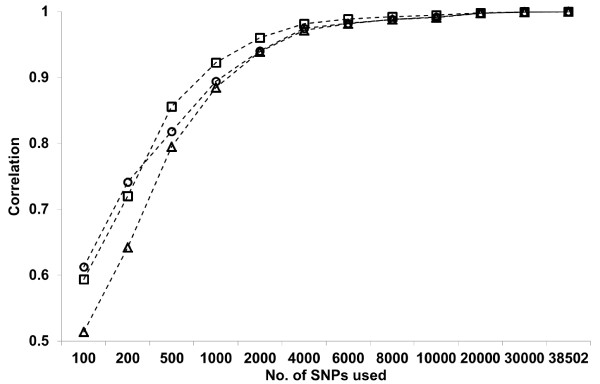
**Changes in *r***_***D***_**, *r***_***N***_**and *r***_***A***_**with increasing density of SNPs used to construct G matrix.** Circles: *r*_*D*_; triangles: *r*_*N*_; squares: *r*_*A*_.

Table 1 also shows correlations between the diagonal (*r*_*D*_), upper triangular (*r*_*N*_) and all the elements (*r*_*A*_) of a given **G** matrix, and the corresponding elements of the **G** matrix constructed using all available SNPs. The *r*_*N*_ was 0.51, 0.79, 0.88, 0.94, 0.97 and 0.99 using 100, 500, 1,000, 2,000, 4,000 and 8,000 SNPs, respectively. The changes in *r*_*D*_, *r*_*N*_ and *r*_*A*_ with increasing SNP density are depicted in Figure 2. A correlation of 0.73 was observed between *r*_*N*_ and mean *r*^*2*^, showing a very high linear relationship especially for SNP sets with smaller numbers. Analysing data from the 50K chip for 1,707 AI sires, along with the records of 698 steers of the Angus breed, Rolf *et al.*[17] showed that the average correlation of upper triangular elements between **G** matrices constructed from all available SNPs, and from its subset of SNPs selected randomly, reached nearly 0.8 using 1,000 SNPs, and exceeded 0.9 using 2,500 SNPs, suggesting that 2,500–10,000 SNPs distributed throughout the genome are required to robustly estimate a **G** matrix for feed efficiency traits with heritability ranging from 0.09–0.14. The changing patterns in Figure 2 are similar to those of [18], although we used a scheme of equally-spaced selection in the number of SNPs. Compared with [17], however, the correlation (*r*_*N*_) in the current study reached 0.9 using a lower number of SNPs and 0.99 using 8,000 SNPs, which would, at least in part, be due to a smaller effective size of the Japanese Black population.

### Genetic variance explained

**Figure 3:**
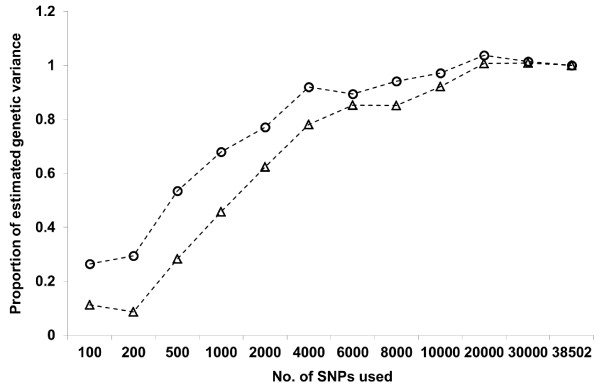
**Changes in proportions of estimated genetic variances in model 1, with increasing density of SNPs used to construct G matrix.** Circles: carcass weight; triangle: marbling score.

Results of variance component estimation for carcass weight and marbling score, using all available SNPs or subsets, by the conventional linear model (model 1) are presented in Tables 2 and 3, respectively. Figure 3 depicts the changes in proportions of estimated genetic variances for both traits. Genetic and residual variances, or $\sigma_{g}^{2}$ and $\sigma_{e}^{2}$, estimated with the **G** matrix using all available SNPs, were 1096.3 and 928.1 kg^2^ for carcass weight and 8.30 and 3.81 score^2^ for marbling score, respectively, which resulted in heritability estimates of 0.54 and 0.68, respectively. These estimates of heritability were similar to those previously estimated in the Japanese Black population using pedigree information [18], although we note that our estimate for marbling score might be somewhat overestimated because of the distribution of the records used. Heritability of human height was estimated to be 0.45 using 565,040 autosomal SNPs from over 10,000 unrelated individuals [19], which is lower than the estimates of 0.8–0.9 reported in previous family and twin studies [20]. The effective population size of humans was estimated to be 10,000 [21], and therefore, for human polygenic traits like height, many more SNPs for a much higher LD level with causative variations would be needed to capture the total genetic variation. In contrast, the effective population sizes of cattle breeds would be much smaller, usually 100 or lower. In the case of Japanese Black cattle, where the effective size is only about 30 [8], it is likely that a large part of the genetic variance for the carcass traits studied here could be captured by using all available SNPs within the 50K chip.

**Table 2:** Variance components estimated with model 1 for carcass weight

**No. of SNPs selected**	$\sigma_{e}^{2}{\left(kg^{2}\right)}^{*1}$	$\sigma_{g}^{2}{\left(kg^{2}\right)}^{*1}$	$\sigma_{p}^{2}{\left(kg^{2}\right)}^{*1}$	$\sigma_{g}^{2}/\sigma_{p}^{2}$
100	1798.9 (193.8) ± 90.7	289.9 (26.4) ± 78.1	2088.8 (103.2) ± 112.9	0.14 ± 0.03
200	1737.7 (187.2) ± 91.1	322.4 (29.4) ± 76.1	2060.0 (101.8) ± 107.7	0.16 ± 0.03
500	1447.0 (155.9) ± 86.0	586.8 (53.5) ± 102.5	2033.7 (100.5) ± 109.3	0.29 ± 0.04
1,000	1290.3 (139.0) ± 90.1	745.1 (68.0) ± 121.9	2035.4 (100.5) ± 111.3	0.36 ± 0.05
2,000	1168.5 (125.9) ± 96.6	844.9 (77.1) ± 135.8	2013.4 (99.5) ± 110.6	0.42 ± 0.05
4,000	1025.2 (110.5) ± 107.7	1008.6 (92.0) ± 160.7	2033.8 (100.5) ± 114.7	0.49 ± 0.06
6,000	1028.9 (110.9) ± 107.5	980.0 (89.4) ± 155.5	2009.0 (99.2) ± 111.2	0.49 ± 0.06
8,000	992.1 (106.9) ± 113.0	1032.1 (94.1) ± 165.5	2024.2 (100.0) ± 113.3	0.51 ± 0.06
10,000	956.4 (103.1) ± 112.5	1065.1 (97.2) ± 166.7	2021.5 (99.9) ± 113.5	0.53 ± 0.06
20,000	895.6 (96.5) ± 117.1	1137.0 (103.7) ± 176.7	2032.6 (100.4) ± 115.5	0.56 ± 0.07
30,000	915.8 (98.7) ± 117.2	1112.6 (101.5) ± 174.2	2028.5 (100.2) ± 114.3	0.55 ± 0.07
38,502	928.1 (100) ± 117.6	1096.3 (100) ± 173.5	2024.4 (100) ±113.7	0.54 ± 0.07
Imp1^*2^	867.2 (93.4) ± 119.7	1166.6 (106.4) ± 180.3	2033.8 (100.5) ± 115.6	0.57 ± 0.07
Imp2^*2^	931.1 (100.3) ± 118.0	1093.9 (99.8) ± 173.5	2025.0 (100.0) ± 113.7	0.54 ± 0.07

*^1^Values in parentheses represent the percentage relative to the estimate obtained with model 1 incorporating the **G** matrix constructed using all available SNPs.

*^2^Imp1 and imp2: 38,502 SNP genotypes imputed from 4,000 and 10,000 SNPs, respectively.

**Table 3:** Variance components estimated with model 1 for marbling score

**No. of SNPs selected**	$\sigma_{e}^{2}{\left(\mathrm{scor}e^{2}\right)}^{*1}$	$\sigma_{g}^{2}{\left(\mathrm{scor}e^{2}\right)}^{*1}$	$\sigma_{p}^{2}{\left(\mathrm{scor}e^{2}\right)}^{*1}$	$\sigma_{g}^{2}/\sigma_{p}^{2}$
100	10.68 (280.4) ± 0.55	0.93 (11.3) ± 0.38	11.62 (95.9) ± 112.9	0.08 ± 0.03
200	10.81 (283.9) ± 0.57	0.72 (8.7) ± 0.35	11.54 (95.3) ± 107.7	0.06 ± 0.03
500	9.27 (243.4) ± 0.55	2.35 (28.3) ± 0.55	11.63 (96.0) ± 109.3	0.29 ± 0.04
1,000	8.01 (210.4) ± 0.57	3.80 (45.7) ± 0.73	11.81 (97.5) ± 111.3	0.32 ± 0.05
2,000	6.64 (174.2) ± 0.57	5.18 (62.4) ± 0.82	11.82 (97.6) ± 110.6	0.44 ± 0.05
4,000	5.40 (141.8) ± 0.59	6.49 (78.2) ± 0.92	11.89 (98.2) ± 114.7	0.54 ± 0.06
6,000	4.85 (127.2) ± 0.60	7.07 (85.2) ± 0.97	11.92 (98.4) ± 111.2	0.59 ± 0.06
8,000	4.86 (127.7) ± 0.62	7.07 (85.1) ± 0.98	11.93 (98.5) ± 113.3	0.59 ± 0.06
10,000	4.36 (114.5) ± 0.63	7.65 (92.2) ± 1.03	12.01 (99.2) ± 113.5	0.63 ± 0.06
20,000	3.74 (98.1) ± 0.64	8.36 (100.7) ± 1.08	12.10 (99.9) ± 115.5	0.69 ± 0.06
30,000	3.77 (98.8) ± 0.66	8.37 (100.8) ± 1.10	12.13 (100.2) ± 114.3	0.69 ± 0.06
38,502	3.81 (100) ± 0.66	8.30 (100) ± 1.09	12.11 (100) ± 113.7	0.69 ± 0.06
Imp1^*2^	4.11 (107.9) ± 0.68	8.01 (96.6) ± 1.09	12.12 (100.1) ± 115.6	0.66 ± 0.06
Imp2^*2^	3.91 (102.7) ± 0.66	8.19 (98.7) ± 1.09	12.10 (99.9) ± 113.7	0.67 ± 0.06

*^1^Values in parentheses represent the percentage relative to the estimate obtained with model 1 incorporating the **G** matrix constructed using all available SNPs.

*^2^Imp1 and imp2: 38,502 SNP genotypes imputed from 4,000 and 10,000 SNPs, respectively.

As expected, as the number of SNPs used became higher, estimated residual and genetic variances gradually decreased and increased, respectively. This is mainly because the higher the SNP marker density, the higher the LD levels between SNP markers and true QTL regions. For instance, in the case of carcass weight, correlations between mean *r*^*2*^ and the estimates of $\sigma_{e}^{2}$ and $\sigma_{g}^{2}$ in model 1 were −0.79 and 0.80, respectively. For both carcass traits, considering standard errors, a largely constant value of phenotypic variance $\left(\sigma_{p}^{2}\right)$ was obtained, even with the different numbers of SNPs used. It was also observed that the value of genetic variance per SNP became larger when fewer SNPs were used (data not shown), which would be partly due to the additional variance explained by the correlated effect of SNPs around those used to construct the **G** matrix. However, the proportion of genetic variance explained by SNPs decreased slightly with an increase from 4,000 to 6,000 and from 6,000 to 8,000 SNPs, for carcass weight and marbling score, respectively (Figure 3). This could be interpreted partly as a reflection of the genetic background and architecture, or the distribution of real QTL regions and their effects relevant to each trait, in Japanese Black cattle.

For carcass weight, approximately 90 and 97% of the genetic variance estimated with the **G** matrix using all available SNPs was obtained using 4,000–6,000 and 10,000 SNPs, respectively. For marbling score, the proportion of the genetic variance accounted for by a given number of SNPs was consistently low when compared with carcass weight, particularly when a relatively small number of SNPs were used. This finding may indicate that the degree of marbling is controlled by only QTLs with relatively small effects, compared with the carcass weight. In fact, three QTLs for carcass weight, called CW-1, -2 and -3, have been identified in genome-wide association studies (GWAS), in which their allele substitution effects were relatively large [22-25], whereas no such QTLs have been detected for the degree of marbling until now. Using 10,000 SNPs, however, as much as 92% of genetic variance in marbling score was accounted for in this study.

### Accuracy of genomic estimated breeding value

Correlations and linear regressions on GEBVs obtained with the different densities of SNPs used are shown in Table 4. When 4,000 and 10,000 SNPs were used in model 1, the correlations between the GEBVs and those obtained using all available SNPs were both 0.99 for carcass weight and 0.98 and 0.99 for marbling score, respectively, with the corresponding linear regression coefficients of 0.94 and 0.98 for the former trait and 0.82 and 0.94 for the latter trait. This showed a trend of underestimation of GEBVs with a lower number of SNPs used, particularly for the latter trait. The different levels of underestimation of GEBVs could be because of different genetic architectures of the two traits. As stated previously, while three QTLs with relatively large effects on carcass weight in Japanese Black cattle have been found [22-25], no such QTLs have been found for degree of marbling. Thus, considering the results of the estimated genetic variances, the lower underestimation of GEBVs observed for carcass weight relative to marbling score might reflect the observation that relatively larger effects of SNPs linked to the carcass weight QTLs could be better captured, even with a lower number of SNPs.

**Table 4:** Correlation between and linear regression of GEBVs obtained with model 1 using a given SNP set and all available SNPs

**No. of SNPs selected**	**Correlation coefficient**		**Regression coefficient**	
	**Carcass weight**	**Marbling score**	**Carcass weight**	**Marbling score**
100	0.64	0.48	0.34	0.12
200	0.71	0.53	0.40	0.10
500	0.86	0.75	0.64	0.32
1,000	0.92	0.87	0.75	0.48
2,000	0.96	0.94	0.84	0.66
4,000	0.99	0.98	0.94	0.82
6,000	0.99	0.99	0.94	0.88
8,000	0.99	0.99	0.96	0.88
10,000	0.99	0.99	0.98	0.94
20,000	0.99	0.99	1.02	1.01
30,000	0.99	0.99	1.01	1.00
Imp1^*^	0.99	0.99	1.03	0.96
Imp2^*^	0.99	0.99	1.00	0.99

*Imp1 and imp2: 38,502 SNP genotypes imputed from 4,000 and 10,000 SNPs, respectively.

### Use of imputed genotype information

**Figure 4:**
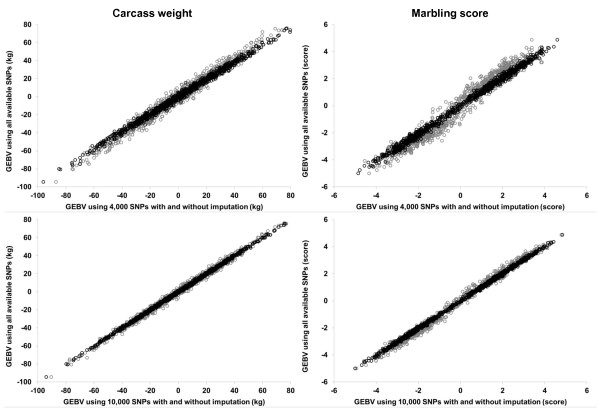
**Scatter plots for GEBVs obtained with model 1 using 4,000 (top panels) or 10,000 (bottom panels) SNPs and those using all available SNPs, for carcass weight (left panels) and marbling score (right panels) with and without imputation.** Black circles: with imputation; gray circles: without imputation.

Accuracy of imputation, expressed as the percentage of correctly predicted genotypes, was 93.4 ± 2.5 and 97.4 ± 1.2% (average ± standard deviation) for 38,502 genotypes imputed from 4,000 and 10,000 SNPs, respectively. Variance components estimated using the imputed genotype data are shown in Tables 2 and 3. Scatter plots of the GEBVs obtained for carcass weight and marbling score against those obtained using all the available SNPs without imputation are shown in Figure 4. Use of the imputed data resulted in a similar level of estimated variances as the level obtained using all the SNPs without imputation. Correlations between the GEBVs obtained with imputation and those obtained from all the SNPs without imputation were higher than 0.99 for both the traits. Imputation of SNP genotypes from low density to high density is now a standard procedure for using low-density marker panels in GS schemes [5,26]. Our results using the imputed SNP information support the use of the imputation from low-density marker panels.

### Estimation using a threshold model

**Figure 5:**
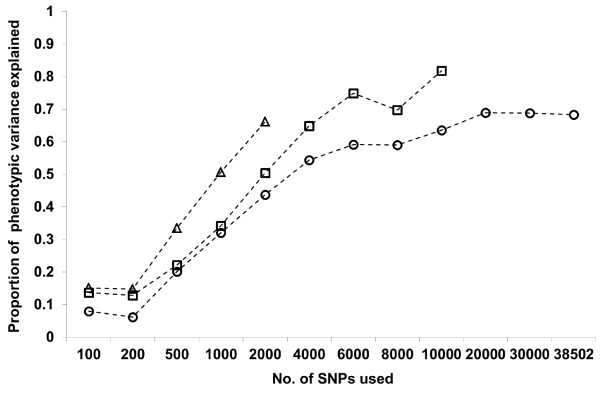
**Changes in proportions of phenotypic variances explained with model 1 and 2 for marbling score, with increasing density of SNPs used to construct G matrix.** Circles: model 1; triangle: model 2 (binary); square: model 2 (categorical with 11 categories).

The proportions of genetic variances to phenotypic variances in the underlying scale estimated for marbling score using the threshold model (model 2) are presented in Table 5. For both the binary and more categorical treatments, the estimations were successful only when relatively small numbers of SNPs were used. The changes in the estimated proportions, relative to the proportions estimated using model 1, are depicted in Figure 5. The values of some correlations between GEBVs obtained with models 1 and 2 are listed in Table 6. It has been noted that generalized linear animal models are plagued by extremely slow mixing in implementations of Markov chain Monte Carlo methods [27]. For both the successful and unsuccessful estimations, a single chain of 10,000,000 samples was run with the first 3,000,000 samples being discarded. The results showed that the estimates presented in Table 5 were not substantially different from those obtained, while there was still no convergence for any of the unsuccessful cases. The failures of the estimations using the larger numbers of SNPs may be attributed largely to the limited number of animals used in this study.

**Table 5:** Proportion of genetic to phenotypic variances estimated with model 2 for marbling score

**No. of SNPs selected**	Binary*	Categorical*
100	0.15 ± 0.04	0.14 ± 0.03
200	0.15 ± 0.04	0.13 ± 0.03
500	0.33 ± 0.06	0.22 ± 0.04
1,000	0.51 ± 0.08	0.34 ± 0.06
2,000	0.66 ± 0.08	0.50 ± 0.06
4,000	-	0.65 ± 0.07
6,000	-	0.75 ± 0.08
8,000	-	0.70 ± 0.07
10,000	-	0.82 ± 0.07

^*^Binary: treated as a binary trait; Categorical: treated as a categorical trait with 11 categories.

**Table 6:** Correlations between GEBVs obtained with models 1 and 2 using a given SNP set and all available SNPs for marbling score

**No. of SNPs selected**	**Model 1 with all 38,502 SNPs**		**Model 1 with each SNP set**		**Between binary and categorical**
	Binary^*^	Categorical^*^	**Binary**	**Categorical**	
100	0.46	0.47	0.96	0.96	0.87
200	0.52	0.53	0.95	0.95	0.84
500	0.72	0.74	0.97	0.97	0.89
1,000	0.83	0.84	0.96	0.97	0.88
2,000	0.90	0.92	0.96	0.97	0.88
4,000	-	0.95	-	0.97	-
6,000	-	0.96	-	0.96	-
8,000	-	0.96	-	0.96	-
10,000	-	0.96	-	0.96	-

^*^Binary: treated as a binary trait; Categorical: treated as a categorical trait with 11 categories.

The proportions of the genetic to the phenotypic variances estimated with model 2 were observed to be consistently larger than the corresponding estimates with model 1. The estimates of heritability for marbling score of Japanese Black cattle reported in the literature [18] indicate that the genetic variances in the underlying scale obtained with model 2 in this study may be somewhat inflated; for instance, 0.66 with 2,000 SNPs for the binary case and 0.82 with 10,000 SNPs for the case of 11 categories . However, for a given set of selected SNPs, the correlations between GEBVs obtained with model 2 and GEBVs obtained with model 1 using all the SNPs were found to be similar to the corresponding values in the analyses with model 1. The correlations between the GEBVs obtained with both the models using a given set of SNPs were very high overall. These correlations between the GEBVs would generally support the validity of the results for marbling score obtained with model 1.

### Overall discussion

Equally-spaced panels with various densities are already used in many situations. Such panels have the advantage of being applicable irrespective of trait and population, for which the density of SNPs plays an important role in GE and GS according to the extent of LD between SNP markers and real QTLs. At this stage, the 50K chip is most commonly used. Using higher density-panels, such as the 50K and 770K, may account for a high to very high proportion of genetic variation. In addition, as shown also in this study, use of genotypes imputed from low-density to high-density can take account of genetic variance largely. However, while increasing density of SNPs used could increase the extent of the LD, it could also increase the number of uninformative and collinear SNPs [28]. Thus, for robust prediction it is important to exclude collinear nuisance SNPs, since their inclusion in the analyses may increase error and sampling variances in estimation of SNP effects on the training population or allow a single QTL to be attributed to a number of highly correlated SNPs, which would be likely to reduce the predictability of GEBVs and its persistence across generations. Schulz-Streeck *et al.*[29] confirmed this by simulation, finding that excluding the markers with negligible or inconsistent effects by pre-selection increases the accuracy of GE.

From this perspective, even the 3K chip has been suggested to be a useful tool in dairy GE [30]. Also, evaluating the predictive ability of subsets of SNPs, Moser *et al.*[31] concluded that accurate GE of Holstein bulls and cows can be accomplished with 3,000–5,000 equally spaced SNPs. From the viewpoint of the relationship of *r*^2^ to the accuracy of GEBV, a simulation study showed that while the accuracy of GEBVs for unphenotyped animals ranged from about 0.65, for the mean *r*^*2*^ of 0.1 between adjacent markers, to more than 0.80, for the mean *r*^*2*^of 0.2, the accuracy for phenotyped animals exceeded 0.8, with a mean *r*^*2*^ of 0.1, with heritability of 0.5 [32]. The mean of *r*^*2*^ was almost 0.1 when 10,000 SNPs were used in the current study (Table 1), and the level of heritability estimated using all available SNPs was more than 0.5 for both the traits (Tables 2 and 3). Therefore, using 10,000 equally-spaced SNPs, which is relatively few compared with all available SNPs in the 50K chip, might be sufficient to cover both of the carcass traits, even in validation and application populations. Moreover, as far as genetic evaluation for ranking animals is concerned, the current results might suggest a possibility of using 4,000–6,000 equally-spaced SNPs for these carcass traits in the Japanese Black population in Japan, since the downward bias in GEBV values observed in this study with lower densities of SNPs would not substantially influence the ranking of animals. Such lower density panels could be used practically in pre-selection, especially of young breeding females whose number in the population is definitely high. This could be beneficial, even with the current degree of accuracy, in dramatically reducing the total cost of the genetic evaluation, since carcass traits are usually measured only on their relatives. If necessary, the imputation of SNP genotypes from the lower density panels to higher density panels, as indicated in [33], could help to achieve an additional increase in the accuracy of GE. On the other hand, young breeding bulls to be selected as future elite AI sires should be genotyped with a high-density panel for more reliable GE and GS, since the contribution of elite AI sires to genetic improvement is significant.

There are several reports on ways of choosing unequally-spaced SNPs, as well as equally-spaced SNPs as a subset, particularly in a relatively low-density panel, and on the utility of low-density marker panels (e.g., [34-36]). Of these ways, choosing SNPs ranked highly in the magnitude of the absolute value of estimated effect is typical. In most cases, prediction of GEBVs with high-ranking SNPs is somewhat more accurate and reliable than with equally-spaced SNPs, when the same number of SNPs is used in the prediction (e.g., [35,36]). For Japanese Black cattle, only one previous study, conducted from the viewpoint of GE, performed the estimation of variance for carcass traits [37]. This study used 50K SNP genotype data from 673 steers to simply perform linear regression analysis of each SNP for each trait, and subsets of SNPs with various significance levels for the association with each trait were used to account for variances. Including this study, however, use of SNPs ranked highly based on certain criteria would generally be applicable only to a particular trait and population. One approach is to integrate the optimal subset of the SNPs for each of several important traits into one set, which is as cheap as possible to use in the target population, as ordinary selection is often implemented for certain multiple traits, although this strategy still requires the re-selection of SNPs with process of generation. While use of an equally-spaced SNP panel deals with all the genome regions, according to density, a trait-specific panel would frequently deal with only parts of the genome. Thus, a compromise plan, as suggested by [34], might be practical, in which a large part of the whole SNP set is composed of equally-spaced SNPs, and SNPs that are in high LD with the causative variants are also included. An example of the latter SNPs for carcass weight in Japanese Black cattle is those linked tightly with CW-1, -2 and -3, found by [22-25]. In addition, since pedigree data are important information irrespective of traits [35], if deep and wide pedigree data can be combined with a SNP set, as mentioned above, more effective GE and GS might be possible. More studies of sophisticated approaches to construct an optimal SNP set for valid and cost-effective GE of carcass traits in beef cattle are required.

In this study, we employed a scheme of equally-spaced selection of SNPs to investigate carcass weight and marbling score, which are representative traits for carcass quantity and meat quality, respectively. We have provided important basic information on the relationships between SNP marker density and genetic variance explained and accuracy and bias of GEBVs obtained. However, as the size of the dataset available in this study was limited, all of the available animals were used in the estimation analyses. In the analyses, the number of animals available (about 900) was well exceeded by the number of SNPs in most settings of SNP selection and use. Thus, we note that the genetic variance explained and the accuracy of GEBVs obtained in the current study may be somewhat inflated, relative to those values obtained using many independent validation animals. Therefore, further research is needed to confirm the current findings, especially from the perspective of prediction, and accumulating a much larger volume of relevant data.

## Conclusions

To our knowledge, this is the first study reporting the level of whole genome LD in Japanese Black cattle, using about 40,000 genome-wide SNPs, as well as the effects of their equally-spaced subsets on the elements of the **G** matrix, the degree of genetic variance explained and accuracy of GEBVs for carcass weight and marbling score, which are representative traits for carcass quantity and meat quality, respectively. Our study revealed that the Japanese Black is likely to be in a breed group with a relatively high extent of whole genome LD, and that the degree of marbling is controlled by only QTLs with relatively small effects, compared with carcass weight. The possibility of effective GE with at least 4,000 equally-spaced SNPs was suggested for these traits.

## Methods

### Ethics statement

Animal care and use was according to the protocol approved by the Shirakawa Institute of Animal Genetics Animal Care and Use Committee, Nishigo, Japan (ACUCH21-1).

### Phenotype data

**Figure 6:**
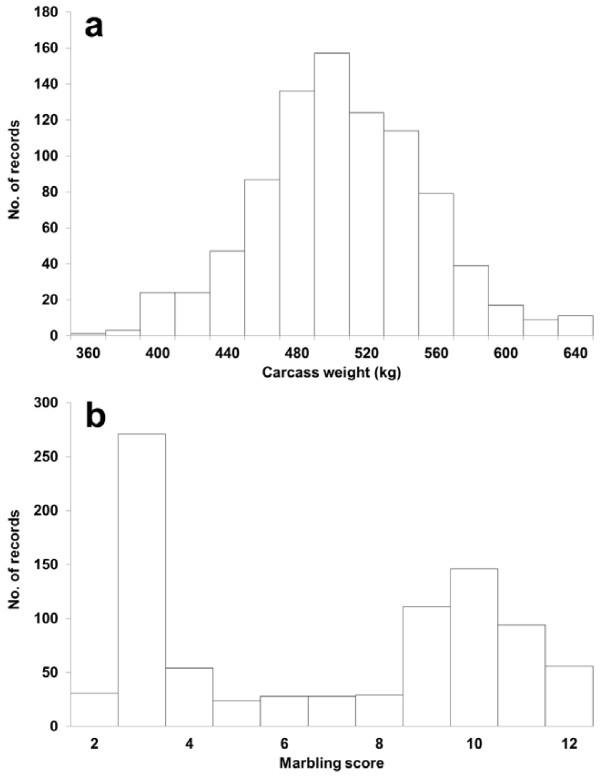
Distribution of carcass weights (a) and marbling scores (b).

Cold carcass weights and marbling scores of 872 Japanese Black fattened steers, whose ages ranged between 15.3–43.0 months, were used for the current analyses. These records were collected from 2000–2009 at two large meat markets in Japan, namely Tokyo Metropolitan Central Wholesale Market and Osaka Municipal South Port Wholesale Market. Marbling score is the degree of marbling, ranging from null (1) to very abundant (12), assessed on the ribeye of the carcass dissected at the sixth and the seventh rib section, according to the Japan carcass grading standards [38]. The distributions of carcass weight and marbling score are shown in Figure 6. The mean (± standard deviation) was 496.6 (± 48.0) kg for carcass weight and 6.8 (± 3.5) for marbling score.

### Genotype data

DNA samples were extracted from perirenal adipose tissues. Sample DNA was quantified and genotyped using the BovineSNP50 BeadChip (hereafter referred to as the 50K chip). The 50K assay contains 54,001 SNPs with an average probe spacing of 51.5 kb and a median spacing of 37.3 kb. A total of 38,502 SNPs were included in the statistical analyses, based on the following criteria: MAF and genotype call rates were larger than 0.01 and 0.95, respectively, were in Hardy-Weinberg equilibrium (*p* > 0.001) and had position information. As a few percent of genotype data was missing, missing genotype filling was conducted using “Beagle 3.3.2” package [39].

### Statistical analyses

Data were analysed using a following linear model (denoted as model 1):

y=Xb+g+e

where **y** is the vector of records, **b** is the vector of fixed discrete effects of market and year at slaughter and the continuous effects of the linear and quadratic covariates of month of age at slaughter, **g** is the vector of additive genetic effects being assumed to follow $N\left(0,\;G\sigma_{g}^{2}\right)$ with the genetic (polygenic) variance and the genomic relationship matrix represented by $\sigma_{g}^{2}$ and **G**, respectively, **e** is the vector of residuals assumed to follow $N\left(0,\;I\sigma_{e}^{2}\right)$ with the residual variance and the identity matrix denoted by $\sigma_{g}^{2}$ and **I**, respectively, and **X** is incidence matrix.

Using the SNP genotype data, the **G** matrix was constructed according to [3] by:

$$G=\left(M-2P\right){\left(M-2P\right)}^{\prime }/\left\{2\sum_{i}^{n}p_{i}\left(1-p_{i}\right)\right\}$$

where **M** is the matrix whose row elements include the number of minor alleles in each animal at each SNP locus, **P** is the matrix whose row elements contain the MAF at each SNP locus, *p*_*i*_ is the MAF at the *i*th SNP locus, and *n* is the number of SNPs used.

In this study, 12 different **G** matrices were constructed and employed by selecting from 100 to 30,000 equally-spaced SNPs in number or using all available SNPs. To make the **G** matrices always positive definite, 10^-4^**I** was added to **G** in construction. We note that pedigree information for the animals, consequently the **A** matrix, was not available in this study.

For each of the 12 sets of SNPs, including the set of all available SNPs, the extent of LD was measured by the squared correlation (*r*^*2*^) of the alleles at two loci for all pairs of two adjacent SNPs on all chromosomes [40]. The mean and standard deviation of the distance (*d*) between two adjacent SNPs were also calculated. In addition, correlations between the diagonal, upper triangular, and all the elements of a given **G** matrix, and the corresponding elements of the matrix constructed using all available SNPs, were examined (denoted as *r*_*D*_, *r*_*N*_ and *r*_*A*_, respectively).

To assess the relationships between GEBVs $\left(\hat{g}\right)$ in each model, correlations were computed between GEBVs incorporating the **G** matrix constructed using all available SNPs, and those incorporating the **G** matrix using a given number of SNPs. Also, linear regressions were fit, where the dependent variables were GEBVs incorporating the **G** matrix constructed using a given number of SNPs, and the independent variable was GEBVs obtained using all available SNPs.

Additionally, choosing the two lower-density subsets, or those of 4,000 and 10,000 SNPs, we attempted to carry out the genotype imputation with “Beagle 3.3.2” from those to all the 38,502 SNPs, in which as a reference, phased haplotype data of 494 animals not having records of both the traits whose data were collected at the same two markets as the 872 animals. Then, using the imputed data, the analyses with model 1 were also conducted.

Furthermore, the distribution of marbling score was obviously far from a normal distribution, as shown in Figure 6. Then, for this trait, a threshold model (model 2) was also fit, as follows:

η=Xb+g+e

where **η** is the vector of unobserved variables in the underlying scale, assuming that $\sigma_{e}^{2}=1$. Two sorts of analysis were conducted regarding the outward phenotype as either a binary trait in which the observed scores 2–6 and 7–12 were each classified into one class, or an ordered categorical trait using actually observed scores.

All the parameters in model 1 were estimated via the Bayesian framework using Gibbs sampling in “BLR” package under R environment [41,42]. A flat prior distribution was used for the nuisance parameters (**b**), and multivariate normal distributions were employed as priors for the additive genetic effects. As prior distributions for $\sigma_{g}^{2}$ and $\sigma_{e}^{2},$ independent scaled inverted chi-square distributions were used with degree of belief and scale parameters of −2 and 0, respectively, assuming that there was no prior information. The “BGLR” package, or an improved version of the BLR software [43], was used to estimate the parameters in model 2. A single chain of 110,000 samples was run, and the first 10,000 samples were discarded as burn-in. Posterior summaries, or mean and standard deviation here, were computed using a thinning rate of 10.

## Abbreviations

QTLs: Quantitative trait loci; GE: Genomic evaluation; GS: Genomic selection; SNPs: Single nucleotide polymorphisms; LD: Linkage disequilibrium; BLUP: Best linear unbiased prediction; G matrix: Genomic relationship matrix; A matrix: Additive relationship matrix; GEBV: Genomic estimated breeding value; MAF: Minor allele frequencies; AI: Artificial insemination; GWAS: Genome-wide association study.

## Competing interests

The authors declare that they have no competing interests.

## Authors’ contributions

HI, HM and SO conceived of the study. SO carried out the analyses and drafted the manuscript. HI and HM contributed in writing and improving the manuscript. YT, TW, SN and YS participated in the design of the study. All authors read and approved the final manuscript.
